# RNF20-SNF2H Pathway of Chromatin Relaxation in DNA Double-Strand Break Repair

**DOI:** 10.3390/genes6030592

**Published:** 2015-07-14

**Authors:** Akihiro Kato, Kenshi Komatsu

**Affiliations:** Division of Genome Repair Dynamics, Radiation Biology Center, Kyoto University, Yoshida-konoecho, Sakyo-ku, Kyoto 606-8501, Japan; E-Mail: komatsu@house.rbc.kyoto-u.ac.jp

**Keywords:** DNA double-strand break repair, ubiquitylation of histone H2B, RNF20, chromatin relaxation, SNF2H, KAP-1, CHD3.1

## Abstract

Rapid progress in the study on the association of histone modifications with chromatin remodeling factors has broadened our understanding of chromatin dynamics in DNA transactions. In DNA double-strand break (DSB) repair, the well-known mark of histones is the phosphorylation of the H2A variant, H2AX, which has been used as a surrogate marker of DSBs. The ubiquitylation of histone H2B by RNF20 E3 ligase was recently found to be a DNA damage-induced histone modification. This modification is required for DSB repair and regulated by a distinctive pathway from that of histone H2AX phosphorylation. Moreover, the connection between H2B ubiquitylation and the chromatin remodeling activity of SNF2H has been elucidated. In this review, we summarize the current knowledge of RNF20-mediated processes and the molecular link to H2AX-mediated processes during DSB repair.

## 1. Introduction

In eukaryotes, DNA is packaged into condensed chromatin in the form of a nucleosome that consists of two copies of the core histones, H2A, H2B, H3 and H4. Thus, chromatin structures dynamically change to express or repress the genetic information stored in DNA during transcription. Recent studies have revealed that chromatin dynamics in transcription share the common molecular mechanisms in DNA transaction, such as DNA replication and DNA repair. The ubiquitylation of the histone H2B is known to be involved in chromatin dynamics during transcription and lately, a role was also shown in the initiation of homologous recombination through the alteration of the chromatin structure. This is corroborated by evidence that ubiquitylation promotes the accumulation of chromatin remodeling factor, SNF2H, in DNA repair, while it is known to have a decondensing activity during transcription. We first reported the relationship between H2B ubiquitylation and SNF2H in DNA repair in a previous study [1]; thereafter, accumulating evidence by other groups has deciphered the molecular aspects of histone modification in chromatin remodeling. In this article, we summarize the recent advances in understanding the molecular pathway of H2B ubiquitylation for DSB-induced chromatin remodeling.

## 2. Ubiquitylation of Histone H2B

Many post-translational modifications of core histones, including acetylation, methylation, phosphorylation, ubiquitylation, SUMOylation, ADP ribosylation, deimination, and proline isomerization, have been reported [2], while ubiquitylation is limited to the histones such as H2A and H2B [3,4,5]. Yeast histone H2B on lysine 123 (K123) is mono-ubiquitylated with ubiquitin-conjugating enzyme (E2) RAD6 and ubiquitin ligase (E3) BRE1 [6,7,8]. In mammalian cells, the heterodimeric complex of RNF20-RNF40, orthologues of yeast Bre1, mono-ubiquitylates lysine 120 (K120) of H2B in conjunction with its cognate E2 enzyme RAD6 [9,10,11]. It is noted that a heterodimer of two BRE1 paralogues is formed in fission yeast, whereas budding yeast forms a homodimer of single BRE1 [11,12,13]. Although histone H2B is mono-ubiquitylated by other E3 ligases such as BAF250B, Mdm2, BRCA1-BARD1, and MSL2 [14,15,16] and several other residues of H2B, including K34, K46, K108, and K116, are also ubiquitylated [17], mammalian histone H2B at K120 is exclusively mono-ubiquitylated by the RNF20-RNF40 complex [1,18]. Moreover, the ubiquitins bound to H2B are reversibly removed by the deubiquitylating enzymes (DUBs) in cells. Currently, USP3, USP7, USP12, USP22, USP44, USP46, and USP49 in mammals [19,20,21,22,23,24] and Ubp8 and Ubp10 in yeast [25,26] have been implicated as DUBs of ubiquitylated H2B.

Similar to the heterodimeric complexes of RNF20-RNF40, several homodimeric and heterodimeric complex formations have been reported in the RING (really interesting new gene) family of E3 ubiquitin ligase, including cIAP, RNF4, BIRC7, IDOL, CHIP, Prp19, BRCA1-BARD1, Mdm2-MdmX, and RING1B-Bmi1 [27]. The RING family of E3 ligases lose their *bona fide* catalytic center and alternatively, they promote the transfer of ubiquitin from cognate E2 enzyme to their substrates by the activation of the E2 enzyme [27,28]. The RING family of E3 ligase is generally believed to interact with the cognate E2 enzyme through their RING domains, suggesting a critical role of the RING domain in the ubiquitin ligase activity for the activation of E2 enzyme [27,28]. Conversely, the RING domain is also implicated in the formation of the homodimeric and heterodimeric complexes. Both RNF20 and RNF40, as members of the RING family of E3 ligase, have a RING finger domain at their C-terminus, and the RING domain of BRE1 is critical for the ubiquitin ligase activity, similar to other members of the RING family [11,13]. However, the RING domain is dispensable in the interaction with RAD6 cognate enzyme in both RNF20-RNF40 and yeast BRE1 [11,13]. In contrast, this domain is critical for the formation of a heterodimeric complex and stability of each protein [18]. Because the knockdown of either RNF20 or RNF40 induces the degradation of both proteins, their interaction through the RING domain is necessary for their protein stabilities and the resulting ubiquitin ligase activity.

H2B ubiquitylation has been reported in many cellular and biological processes, including DNA replication [29,30], DNA repair [1,18], nucleosome positioning [31], chromatin segregation [32], centromeric chromatin maintenance [33], chromatin boundary integrity [34], RNA processing and export [35,36,37], stem cell differentiation [23,38,39], development [40], exit from mitosis [41], apoptosis [42], tumorigenesis [43,44,45], and viral infection [46,47]. Among them, an accumulating number of reports indicate the strong relationship of H2B ubiquitylation with transcription activation [5,45]. A genome-wide analysis of H2B ubiquitylation using a chromatin immunoprecipitation technique revealed that H2B ubiquitylation is broadly associated with transcribed genes itself but not with the promoter region. This is consistent with the result from the reconstituted transcription assay *in vitro*, showing that transcription elongation is regulated by H2B ubiquitylation [48]. When RNA polymerase II halts at the nucleosome, the histone chaperone FACT (facilitates chromatin transcription) recruits both transcription elongation regulator PAF complex and RNF20/40 heterodimers, which is followed by H2B ubiquityaltion, resulting in the displacement of the histone H2A/H2B dimer to traverse RNA polymerase II through the nucleosome. However, conflicting results for this model have also been reported. Microarray analysis using HeLa cells treated with RNAi revealed that only 6% of the genes were significantly affected by RNF20 depletion, in which approximately half of these were upregulated and the rest were downregulated [43]. This suggests that RNF20-mediated transcription regulation is not simple but rather complicated in cells.

## 3. Chromatin Remodeling with SNF2H

**Figure 1:**
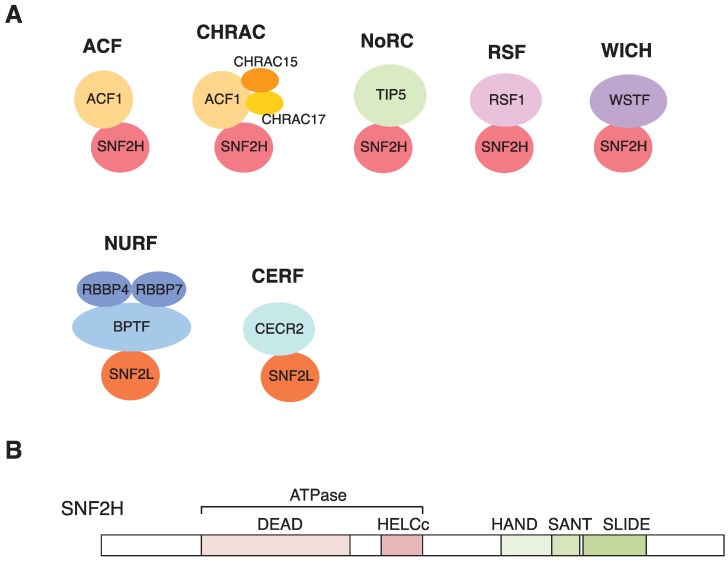
ISWI family of chromatin remodeling complexes in mammal: (**A**) Currently known seven complexes are shown. (**B**) Schematic representation of human SNF2H (sucrose nonfermenting 2 homolog). The ATPase domain is located at N-terminal half of SNF2H. The HSS domain composed of HAND, SANT (Swi3, Ada2, NCoR, TFIIB) and SLIDE (SANT-like ISWI domain) is located at C-terminus.

The proteins that regulate the packaging of DNA into chromatin, termed as chromatin remodeling factors, are involved in many cellular processes dealing with eukaryotic DNA, such as transcription, replication, and repair [49]. There are four ATP-dependent chromatin remodeling families, SWI/SNF, INO80, CHD, and ISWI, all of which are well-conserved through the species [49]. The human ISWI (imitation switch) family represents two kinds of ATPase, SNF2H (also known as SMARCA5) and SNF2L (also known as SMARCA1), which are the orthologues of yeast Isw1 and Isw2, respectively [50]. To date, seven ISWI complexes, including ACF, CHRAC, WICH, RSF, CERF, NoRC, and NURF [51], have been reported in mammals, and all of these have either employed SNF2H or SNF2L as a catalytic subunit of ATPase (Figure 1A). Among the ISWI complexes, ACF, CHRAC, WICH, and RSF containing SNF2H are implicated in DNA repair [52,53,54,55,56], while ACF and WICH also have roles in the maintenance of chromatin structures during DNA replication, where the depletion of SNF2H causes compacting of chromatin [57,58]. Chromatin remodeling factors often associate with histone marks for their localization at the chromatin. Plant homeodomain (PHD) fingers and chromodomains often exist in chromatin remodeling families, and interact with methyl-lysine and acetyl-lysine of histones, respectively [59,60]. Although SNF2H does not have these domains, it is plausible that SNF2H indirectly interacts with modified histones through other accessary subunits containing these domains. Indeed, TIP5, the binding partner of SNF2H in the NoRC complex, has a PHD finger and bromodomain within the structure, and they cooperatively interact with acetylated histone H4 Lys16 (H4K16ac) for rDNA silencing [61]. Similarly, BPTF, the subunit of SNF2L-containing NURF complex, interacts with H3K4me3 and H4K16ac by its PHD finger and bromodomain and recruits NURF complex to transcription sites for its chromatin remodeling [62,63,64]. In DSB repair, ACF1 has been implicated in recruiting SNF2H to DSB sites via an interaction between its PHD domain and trimethylated histone H3 at Lys9 (H3K9me3) (see below).

SNF2H contains an ATPase domain at the N-terminus, which is composed of two conserved regions, DExx and HELICc, termed the DEAD/H superfamily; hence, it can accomplish chromatin remodeling using the energy of ATP hydrolysis [49,65,66]. On the other hand, SNF2H represents the characteristic domains at the C-terminus, which consists of three domains, HAND, SANT (Swi3, Ada2, NCoR, TFIIB), and SLIDE (SANT-like ISWI), termed the HSS domain (Figure 1B). These domains play an important role in nucleosome spacing by binding to a histone tail and DNA [49]. In Drosophila ISWI, X-ray crystallography showed that the SANT domain interacts with histones, and the SLIDE domain binds to nucleosomal DNA [67]. Similarly, the HAND domain of yeast ISW2 interacts near the entry/exit site of the nucleosome, and the SLIDE domain associates with linker DNA [68]. The mutation experiments of the SANT-SLIDE domains suggest that they help to guide the movement of the linker DNA into the nucleosome for the directionality and efficiency of nucleosome sliding along DNA [69,70]. Thus, the ISWI family would achieve either chromosome relaxation or chromatin assembly by the nucleosome sliding activity and result in the optimized nucleosome spacing using the HSS domain at the C-terminus.

## 4. Molecular Link between H2B Ubiquitylation and SNF2H in DSB Repair

Among the many types of DNA damage, DSBs are the most serious lesions. Although DSBs are generated during replication stress and genetic rearrangements of immunoglobulin and T cell receptor genes, ionizing radiation (IR) is known to efficiently elicit DSBs. Once DSBs are induced, they are detected by cellular machinery and rejoined by either of the two major repair pathways, homologous recombination (HR) repair and non-homologous end joining (NHEJ), so that cells can maintain genome integrity. A cellular response to IR occurs immediately after DSB generation because phosphorylation of the histone variant H2AX (so-called γH2AX) can be observed within seconds following IR exposure. ATM (Ataxia telangiectasia mutated) is a major regulatory kinase in DSB signal transduction [71] and phosphorylates more than 700 substrates [72], including H2AX. This ATM activation requires a protein complex consisting of MRE11, RAD50, and NBS1, termed the MRN complex [73,74]. The defect in NBS1 causes enhanced cell killing because of the impaired repair of HR and NHEJ and genomic instability after IR exposure [75,76].

The participation of H2B ubiquitylation in the DSB repair pathway was first implicated in yeast. Game *et al.* reported that a yeast bre1 mutant showed high sensitivity to IR and had epistatic interaction with rad51, the key molecule in HR [77]. This suggests that BRE1 functions in the RAD51-dependent HR repair of DSBs, although the molecular mechanisms in HR are largely unknown. Lately, Nakamura *et al.* and Moyal *et al.* independently reported that RNF20 interacts with NBS1 and ATM in human cells [1,18], indicating that H2B ubiquitylation is involved in NBS1-associated HR and NHEJ repair in human cells too. RNF20 interacts with the C-terminus of NBS1, in which several important domains for protein/protein interaction are located, while the binding site of ATM with RNF20-RNF40 has not yet been reported [1,78,79,80]. The depletion of RNF20 by RNAi resulted in high sensitivity to IR and DNA damaging agents such as neocarzinostatin (NCS), mitomycin C, and camptothecin [1,18,81]. Indeed, DSB repair kinetics, monitored by γH2AX, became slower in RNF20-depleted cells. In addition, the genetic knockdown of RNF20 decreased the DSB repair when HR and NHEJ were quantitatively assayed using DR-GFP and pEJ reporter genes. Consistently, RNF20-depleted cells impaired the accumulation of HR factors such as BRCA1, RAD51, and RPA and NHEJ factors such as XRCC4 at the damage sites, exhibiting the roles of RNF20 in the recruitment of repair proteins to DSB sites [1,18,82]. Intriguingly, this RNF20-dependent accumulation of repair proteins requires ATM activation. Moyal *et al.* reported that S172/S553 of RNF20 and S114 of RNF40 are phosphorylated by ATM after DSB generation [18]. These phosphorylations are a pre-requisite of DSB-induced H2B ubiquitylation. The overexpression of the K120R H2B mutant, that lacks the lysine to be ubiquitylated, also significantly reduced HR frequency, accumulation of both HR and NHEJ proteins at the DSB sites, and the resulting enhancement of cell killing after IR exposure. Moreover, the concurrent depletion of RNF20 and overexpression of mutant H2B had no further effect, suggesting that RNF20 functions through a common pathway of DSB repair with that of H2B ubiquitylation.

**Figure 2:**
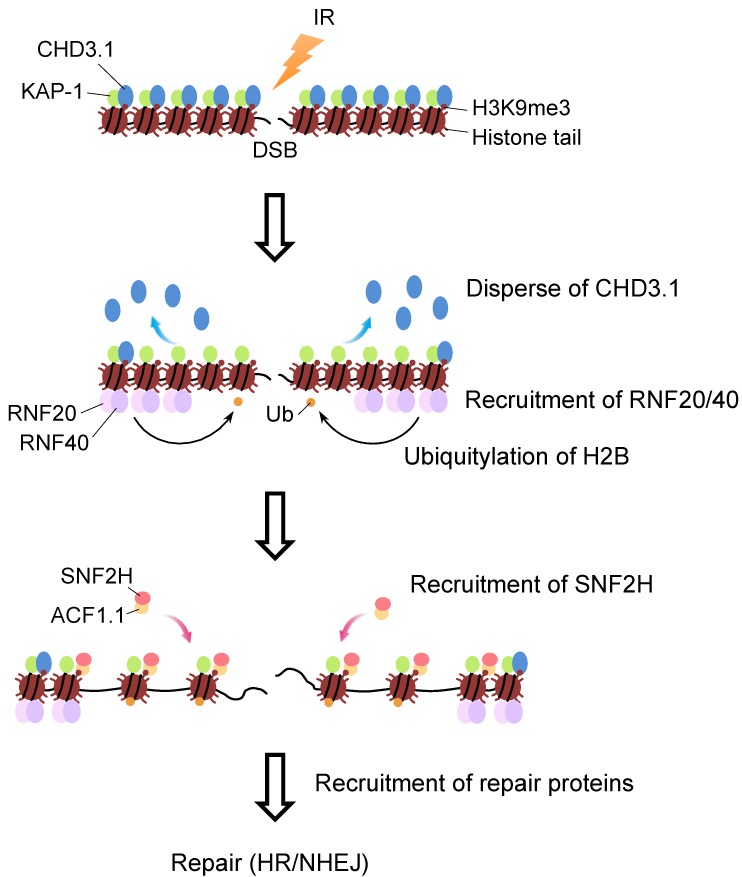
Model of RNF20-SNF2H pathway of chromatin relaxation in DSB repair. Following activation by DSB induction, ATM phosphorylates RNF20 and RNF40. Recruitment of RNF20/40 is carried out via interaction with FACT. Phosphorylated RNF20/40 ubiquitylates H2B at DSB sites. Then, SNF2H is recruited to DSB sites depending on RNF20, SIRT6 and PARP1, and relax compacted chromatin there. Chromatin relaxation by SNF2H requires prior dispersal of CHD3.1 from damage sites. This event is independent from RNF20 pathway. DNA repair proteins such as BRCA1, RAD51, RPA, XRCC4 are able to access damaged DNA after SNF2H-mediated chromatin remodeling.

The roles of RNF20 in the DSB repair pathway is considered to be associated with chromatin relaxation because the defects in the accumulation of the repair protein at the DSB sites were rescued by treatment with several agents that induce chromatin relaxation [1,82]. This is supported by a recent study, which shows that H2B ubiquitylation interferes with chromatin compacting and promotes chromatin accessibility [83]. Furthermore, the chromatin remodeling factor, SNF2H, functions as a downstream protein of RNF20. Because transcription studies have revealed that the ubiquitylation of H2B is followed by SNF2H accumulation, this molecule was a candidate for a factor involved in RNF20-dependent chromatin relaxation. Indeed, several groups reported that the knockdown of SNF2H resulted in similar phenotypes to that of RNF20-depleted cells, such as hypersensitivity to DNA damaging agents and IR, and impaired accumulation of HR and NHEJ proteins at damage sites [1,52,84,85]. Moreover, the concurrent knockdown of RNF20 and SNF2H showed no further reduction of HR frequency than that of a single depletion, indicating epistatic function in the same pathway (Figure 2). Recently, this model of the RNF20-SNF2H chromatin relaxation pathway in DSB repair was elegantly re-confirmed by Klement *et al.* who showed that RNF20 induces local chromatin relaxation in a SNF2H-dependent manner using a visualized LacR/LacO site-specific heterochromatin relaxation assay [54]. They also showed that the catalytic activity of SNF2H and ATM-mediated phosphorylation sites (S172 and S553) of RNF20 are required for NHEJ at the DSB sites, in which the heterochromatin marker H3K9me3 is localized. ACF1, Lan *et al.* showed that a binding partner of SNF2H, also has a crucial role in this pathway because the genetic knockdown of ACF1 showed similar phenotypes to that of the SNF2H mutant [52]. Similar results were also reported by Klement *et al.* who showed that the depletion of SNF2H, ACF1, or RNF20 resulted in the impairment of NCS-induced chromatin relaxation that can be detected by micrococcal nuclease digestion [54]. Therefore, RNF20 and SNF2H function in the same pathway for NHEJ in heterochromatin, although RNF20 seems to have additional roles, including global NHEJ, because knockdown of RNF20 results in the repair defect at a relatively early time point (6 h), whereas knockdown of SNF2H and ACF1 lead to repair defect only at a later time point (16 h) [54]. This is consistent with the observations by Moyal *et al.*, who showed repair defects in RNF20-depleted cells even at an earlier time point (4 h) [18].

It is obvious that both the RNF20-SNF2H pathway and H2AX pathway depends on ATM because both RNF20/RNF40 and H2AX are phosphorylated with ATM kinase after IR exposure. However, it is noteworthy that the concurrent knockdown of RNF20 and H2AX show additive effects on the reduction of HR repair and enhanced IR sensitivity. Similarly, the depletion of H2AX does not affect IR-induced ubiquitylation of H2B and *vice versa* [1]. This evidence suggested that RNF20/40 and H2AX functions in the distinct pathways of the cellular response to IR. Klement *et al.* demonstrated that a CHD family remodeling factor CHD3.1 diffused from heterochromatin after IR exposure, and this dispersal is a prerequisite for chromatin remodeling before ATM- and Artemis-dependent DSB repair [54]. Intriguingly, CHD3.1 functions in the same pathway as H2AX, while it is distinct from the RNF20-SNH2H pathway [54,86,87,88,89]. Both ACF1.1 and CHD3.1 proteins have a common PHD finger to interact with H3K9me3 [54,90] so that both proteins interact with the same histone marker. Interestingly, CHD3.1 enriched at H3K9me3 in heterochromatin was decreased after IR exposure, whereas ACF1.1 was enriched, suggesting a sequential substitution of both proteins at the residue of H3K9me3 after IR exposure. Notably, the phosphorylation of H2AX is a very quick event, whereas the ubiquitylation of H2B and accumulation of RNF20 at damaged sites are much slower [1]. The radiation-induced dispersal of CHD3.1 from heterochromatin requires ATM-mediated phosphorylation of KAP-1 (pKAP-1) at S824 [86,87,88]. This phosphorylation causes the disruption of interactions between SUMOylated KAP-1 and the SUMO-interacting domain of CHD3.1 and thereby detaches from the histone [88]. This dispersal model of CHD3.1 is consistent with the evidence that the NuRD complex containing CHD3.1 participates in the maintenance of transcriptional repression at regions of the genome where DNA is highly methylated and compacted [91]. However, CHD3.1 dispersion is not sufficient for chromatin relaxation, and an additional recruitment of SNF2H to DSB sites is also required, particularly in heterochromatin. Although SNF2H is necessary for chromatin remodeling in both dividing and non-dividing cells, SNF2H is dispensable in dividing CHD3.1-depleted cells. This is because heterochromatic compacting is stably formed in non-dividing cells even after the loss of building factors, whereas dividing cells are unable to form it in the absence of CHD3.1. Thus, decondensing of heterochromatin at DSB sites required two events, in which CHD3.1 initially detaches from H3K9me3 through KAP-1 phosphorylation and SNF2H is subsequently substituted for CHD3.1 at the H3K9me3 site [54].

Recruitment of SNF2H to damage sites has been observed at laser-irradiated regions or densely damaged regions by Fok1 nuclease [52,54,82,84,85]. In both systems, the depletion of RNF20 decreases the accumulation of SNF2H, confirming that RNF20 functions upstream of SNF2H at DSB sites [54,82]. Laser experimentation also showed that PARP and SIRT6 are implicated in this pathway [84,85]; however, it remains inconclusive how these factors are related to each other. Similarly, laser microirradiation revealed that the histone chaperone FACT functions in this pathway as an upstream regulator of RNF20. In transcription, FACT recruits RNF20/40 complex through interaction with PAF1 (RNA polymerase II associated factor) to promote H2Bub-dependent elongation [48]. FACT is a heteromeric dimer of SUPT16H and SSRP1. The depletion of SUPT16H abrogates the accumulation of RNF20 and SNF2H at the damage sites and results in HR defect and hypersensitivity to IR [82,92]. However, RNF20 localization was not affected by PAF1 depletion, even after depletions of ATM and NBS1. Biochemical analysis showed that the RING finger domain at the N-terminus of RNF20 is required for the interaction with the C-terminal region of SUPT16H regardless of transcription, and mutations of this interaction abrogate RNF20 recruitment at the DSB sites. Consequently, RNF20 recruitment to the DSB sites is strongly associated with the activity of the histone chaperone FACT after the generation of DSBs.

## 5. Conclusions

The functional analysis of RNF20 and SNF2H, which had been known in transcription, reveals new roles in chromatin remodeling during DSB repair. From many lines of evidence, the function of RNF20 in DSB repair is mediated by the ubiquitylation of H2B. H2B ubiquitylation and SNF2H appear to sequentially play important roles in ensuring efficient DSB repair. However, how these factors are linked at the molecular level is still unclear. A possible clue that connects these factors directly is histone methylation. It is interesting to note whether H3K9me3-dependent ACF1-SNF2H recruitment would be affected by the H2B ubiquitylation state. The development and analysis of mutants, which harbor a mutation at the ubiquitylation site of H2B (K120), may resolve this question. Although there were technical difficulties in achieving this, because the mammalian genome has at least 17 H2B genes [93], the recently developed technologies of genome editing such as CRISPR-Cas9 and TALEN [94,95,96] would help to overcome this problem in further understanding radiation-induced chromatin reorganization. Since DNA transaction, including transcription and DNA repair, share the common pathway, these mechanical studies would provide useful information in gaining insight into chromatin remodeling in transcription, as well as in DNA repair.
